# Inhibition of Cytochrome P450 Side-Chain Cleavage Attenuates the Development of Mechanical Allodynia by Reducing Spinal D-Serine Production in a Murine Model of Neuropathic Pain

**DOI:** 10.3389/fphar.2019.01439

**Published:** 2019-12-06

**Authors:** Sheu-Ran Choi, Alvin J. Beitz, Jang-Hern Lee

**Affiliations:** ^1^Department of Veterinary Physiology, College of Veterinary Medicine and Research Institute for Veterinary Science, BK21 PLUS Program for Creative Veterinary Science Research, Seoul National University, Seoul, South Korea; ^2^Department of Veterinary and Biomedical Sciences, College of Veterinary Medicine, University of Minnesota, St Paul, MN, United States

**Keywords:** cytochrome P450 side-chain cleavage, D-serine, serine racemase, astrocyte, neuropathic pain

## Abstract

Research indicates that neurosteroids are locally synthesized in the central nervous system and play an important modulatory role in nociception. While the neurosteroidogenic enzyme, cytochrome P450 side-chain cleavage enzyme (P450scc), is the initiating enzyme of steroidogenesis, P450scc has not been examined under the pathophysiological conditions associated with peripheral neuropathy. Thus, we investigated whether chronic constriction injury (CCI) of the sciatic nerve increases the expression of P450scc in the spinal cord and whether this increase modulates serine racemase (Srr) expression and D-serine production contributing to the development of neuropathic pain. CCI increased the immunoreactivity of P450scc in astrocytes of the ipsilateral lumbar spinal cord dorsal horn. Intrathecal administration of the P450scc inhibitor, aminoglutethimide, during the induction phase of neuropathic pain (days 0 to 3 post-surgery) significantly suppressed the CCI-induced development of mechanical allodynia and thermal hyperalgesia, the increased expression of astrocyte Srr in both the total and cytosol levels, and the increases in D-serine immunoreactivity at day 3 post-surgery. By contrast, intrathecal administration of aminoglutethimide during the maintenance phase of pain (days 14 to 17 post-surgery) had no effect on the developed neuropathic pain nor the expression of spinal Srr and D-serine immunoreactivity at day 17 post-surgery. Intrathecal administration of exogenous D-serine during the induction phase of neuropathic pain (days 0 to 3 post-surgery) restored the development of mechanical allodynia, but not the thermal hyperalgesia, that were suppressed by aminoglutethimide administration. Collectively, these results demonstrate that spinal P450scc increases the expression of astrocyte Srr and D-serine production, ultimately contributing to the development of mechanical allodynia induced by peripheral nerve injury.

## Introduction

Evidence to date shows that activation of spinal N-methyl-D-aspartate (NMDA) receptors plays a critical role in the changes in synaptic excitability and the development of neuropathic pain (Woolf and Thompson, 1991; Mao et al., 1992). Intrathecal (i.t.) administration of NMDA receptor antagonists to human significantly reduces neurogenic wind-up pain (Kristensen et al., 1992) as well as allodynia in neuropathic pain patients (Felsby et al., 1996). Competitive and non-competitive NMDA receptor antagonists may have serious side effects that are not seen with glycine site antagonists, and the glycine binding site on the NMDA receptor has become a more attractive pharmacological target (Willetts et al., 1990; Kristensen et al., 1992; Leeson and Iversen, 1994; Ren et al., 2006). NMDA receptors are activated by the binding of glutamate at the glutamate binding site, but also of a co-agonist D-serine or glycine at the glycine binding site (Mothet et al., 2000). D-serine is generated from L-serine by the activity of serine racemase (Srr) in astrocytes; thus, D-serine can play a role as a gliotransmitter released from glia to influence nearby neurons (Wolosker et al., 1999; Wolosker et al., 2002; Ren et al., 2006; Moon et al., 2015). Several reports suggest that peripheral nerve injury increases the expression of Srr in astrocytes and concomitant D-serine production, which play important roles in the functional potentiation of NMDA receptors and the development of peripheral neuropathic pain (Lefèvre et al., 2015; Moon et al., 2015; Choi et al., 2017). Since D-serine can convey nociceptive signaling from glial cells to neurons and change neuronal excitability *via* activation of NMDA receptors, it is important to investigate the regulatory mechanisms underlying the nerve injury–induced increase in the expression and/or activation of astrocyte Srr and accompanying D-serine production.

In the nervous system, “neurosteroids” are synthesized locally rather than in classic steroidogenic organs, and they serve to modulate nervous system activity (Baulieu, 1997; Mellon and Griffin, 2002). The production of endogenous neurosteroids in the spinal cord has been demonstrated by a variety of studies, which showed the presence and activity of several steroidogenic enzymes in the spinal cord (Mensah-Nyagan et al., 2008). The first step in the synthesis of all classes of neurosteroids is the conversion of cholesterol to pregnenolone (PREG). Cytochrome P450 side-chain cleavage enzyme (P450scc) catalyzes this reaction; thus, P450scc plays a crucial role in the initiation of neurosteroids biosynthesis (Le Goascogne et al., 1987; Karri et al., 2007). Then, PREG can be converted to dehydroepiandrosterone (DHEA) by cytochrome P450c17 or to progesterone by 3β-hydroxysteroid dehydrogenase (Compagnone and Mellon, 2000). It has been suggested that neurosteroids are related to the modulation of nociception; thus, neurosteroidogenic enzymes can be potential key therapeutic targets for pain control (Yoon et al., 2010; Porcu et al., 2016; Joksimovic et al., 2018). In previous studies from our laboratories, we suggested that the expression of cytochrome P450c17 is significantly increased in spinal astrocytes following chronic constriction injury (CCI) of the sciatic nerve and inhibition of this enzyme reduces not only the pathophysiological activation of spinal astrocytes but also the development of neuropathic pain (Choi et al., 2019a; Choi et al., 2019c). However, there is limited understanding of the potential role of P450scc, which initiates neurosteroidogenesis, in neuropathic pain under the pathophysiological conditions following peripheral neuropathy.

Thus, we aimed to demonstrate that spinal P450scc-induced initiation of neurosteroidogenesis plays an important role in the development of neuropathic pain and that D-serine could be a potential mediator of this spinal nociceptive transmission. In this regard, we investigated whether: (1) sciatic nerve injury increases the immunoreactivity of P450scc in the spinal cord; (2) i.t. administration of the P450scc inhibitor, aminoglutethimide (AMG), suppresses the CCI-induced mechanical allodynia and thermal hyperalgesia in a mouse model of neuropathic pain; (3) i.t. administration of AMG inhibits the CCI-induced increased expression and/or activation of Srr and D-serine production in the spinal cord; and (4) exogenous D-serine restores the CCI-induced development of the neuropathic pain that was suppressed by the inhibition of P450scc.

## Material and Methods

### Animals

Male Crl:CD1[Institute of Cancer Research (ICR)] mice (20–25 g; 4 weeks old) were obtained from the Laboratory Animal Center of Seoul National University (SNU) in South Korea. Animals were housed under standard laboratory conditions (23±2°C, 12/12 h light/dark cycle) with free access to food and water. All mice were allowed at least 3 days acclimatization period before being used in experiments. The experimental protocols for animal usage were reviewed and approved by the SNU Institutional Animal Care and Use Committee following the National Institutes of Health guide for the care and use of laboratory animals (NIH Publications No. 96-01) revised in 1996.

### Peripheral Nerve Injury Model

Peripheral nerve injury induced by CCI of the sciatic nerve was performed using the method originally described by Bennett and Xie (Bennett and Xie, 1988), while the ligation material was changed from catgut to silk that induces more stable neuropathic pain behaviors in murine CCI models (van der Wal et al., 2015). Briefly, mice were anesthetized with 3% isoflurane in a mixture of N_2_O/O_2_ gas. The right sciatic nerve was exposed and loosely ligated with three ligatures of 6-0 silk thread. Sham surgery was performed by exposing the right sciatic nerve in the same manner, but without ligating the nerve.

### Drugs and i.t. Administration

Drugs used in the present study are as follows: 3-(4-aminophenyl)-3-ethylpiperidine-2,6-dione (AMG, a P450scc inhibitor; 3, 30, 300 nmol); D-serine (500 nmol); and L-serine (D-serine’s enantiomer; 500 nmol). All drugs were purchased from Sigma-Aldrich (St. Louis, MO, USA). The doses of all drugs were selected based on doses previously used in the literature including our previous study (Moon et al., 2015; Choi et al., 2017). D-serine and L-serine were dissolved in physiological saline (5 µl), and AMG was dissolved in a 5 µl mixture of 5% dimethyl sulfoxide and corn oil. All drugs were administered twice a day on postoperative days 0–3 (the induction phase of neuropathic pain) or on postoperative days 14–17 (the maintenance phase of neuropathic pain). Drugs were administered intrathecally using the method described by Hylden and Wilcox (Hylden and Wilcox, 1980) using a 50 µl Hamilton syringe with a 30-gauge needle. Mice were anesthetized with 3% isoflurane in a mixture of N_2_O/O_2_ gas. The insertion of the needle was performed into the L_5–6_ intervertebral space, and a tail flick response was used as an indicator of successful insertion. Each drug was slowly administered for 10 s.

### Nociceptive Behavioral Tests

Mechanical allodynia test was performed using a von Frey filament (North Coast Medical, Morgan Hill, CA) as described previously (Choi et al., 2019a). A von Frey filament with a force of 0.16 g was applied 10 times to the ipsilateral hind paw; then, we recorded the number of paw withdrawal responses. The results were expressed as a percent paw withdrawal response frequency (PWF, %), which represented the percentage of paw withdrawals out of the maximum of 10. Thermal hyperalgesia test was performed using a plantar analgesia meter (Model 390, IITC Life Science Inc., Woodland Hills, CA) as described by Hargreaves et al. with minor modification (Hargreaves et al., 1988). A radiant heat source was applied to the ipsilateral hind paw; then, we measured the paw withdrawal latency (PWL, s) in response to radiant heat. The test was duplicated for the hind paw of each mouse, and the mean withdrawal latency was calculated. A cutoff time of 20 s was used to prevent tissue damage in absence of response. Nociceptive behavioral tests were performed 1 day before surgery to obtain normal baseline values; then, animals were randomly assigned to control and experimental groups. Tests were performed again at 1, 3, 6, 9, 14, and 21 days following surgery in one set of mice or at 3, 6, 9, 14, 15, 17, and 21 days following surgery in a second set of mice. Since we were focusing on the effect of the repeated administration of drugs during the induction and maintenance phase of neuropathic pain, nociceptive behavioral tests were performed at least 4 h post–drug administration to avoid potential effects of a single drug administration. All behavioral analyses were performed blindly.

### Western Blot Assay

The Western blot assay was performed as described previously (Choi et al., 2013; Choi et al., 2019a). Animals were anesthetized with 3% isoflurane in a mixture of N_2_O/O_2_ gas at postoperative day 3 or 17, and sacrificed by transcardial perfusion with calcium-free Tyrode’s solution. The spinal cord segments were extracted by pressure expulsion with air into an ice-cooled and saline-filled glass dish. Next, the spinal cord segments were separated into left (contralateral) and right (ipsilateral) halves under a neurosurgical microscope (Roh et al., 2008). The spinal cord was subsequently further subdivided into dorsal and ventral halves by cutting straight across from the central canal laterally to a midpoint in the white matter. The separated ipsilateral dorsal quadrants of each spinal cord were then used for Western blot analysis. For preparation of Srr cytosolic fraction, the spinal cord dorsal horns were homogenized in lysis buffer A and then centrifuged at 15,000 rpm for 40 min at 4°C. The supernatant was used for Western blot assay. For preparation of total proteins, the spinal cord dorsal horns were homogenized first in lysis buffer A containing 1% Triton X-100 and then centrifuged at 15,000 rpm for 40 min at 4°C. The supernatant was used for Western blot assay. Homogenates (20–25 µg protein) were transferred to nitrocellulose membrane after separation by sodium dodecylsulfate–polyacrylamide gel electrophoresis. The blots were incubated overnight at 4°C with anti-Srr antibody (1:1K, cat# sc-48741, Santa Cruz Biotechnology Inc.) or anti–β-actin antibody (1:5K, cat# sc-47778, Santa Cruz Biotechnology Inc.) and then incubated with a horseradish peroxidase (HRP)-conjugated secondary antibody (1:10K, Santa Cruz Biotechnology Inc.). The intensities of the specific bands were quantified by ImageJ software (ImageJ 1.45s; NIH, USA) and normalized against the loading control. The % change relative to the mean value of control groups was calculated in each group.

### Immunohistochemistry and Image Analysis

Animals were anesthetized at postoperative day 3 or 17, and perfused transcardially with calcium-free Tyrode’s solution followed by 4% paraformaldehyde in 0.1 M phosphate buffer (pH 7.4). The spinal cords were collected, post-fixed overnight, and then placed in 30% sucrose in phosphate-buffered saline at 4°C. Transverse L_4–5_ spinal cord sections (40 µm) were cut using a cryostat (Leica CM1520, Leica Biosystems, Germany). Free floating sections were incubated in blocking solution for 1 h at room temperature and then incubated for 2 days at 4°C with anti–cytochrome P450 (scc) antibody (1:1K, cat# ABS236, Millipore Co.), anti–glial fibrillary acidic protein (GFAP) antibody (1:1K, cat# MAB360, Millipore Co.), anti–neuronal nuclei (NeuN) antibody (1:1K, cat# MAB377, Millipore Co.), anti–ionized calcium-binding adaptor molecule 1 (Iba-1) antibody (1:500, cat# ab5076, Abcam plc.), anti-Srr antibody (1:500, cat# sc-48741, Santa Cruz Biotechnology Inc.), or anti–D-serine antibody (1:500, cat# ab6472, Abcam plc.). Tissue sections are incubated with Alexa 488–conjugated anti-mouse, anti-goat, or anti-rabbit antibody (1:400, Life Technologies), Alexa 555–conjugated anti-mouse or anti-goat antibody (1:400, Life Technologies), or Alexa 568–conjugated anti-rabbit antibody (1:400, Life Technologies) for 1.5 h at room temperature. Fluorescent images were acquired using a confocal microscope (Fluoview 300, Olympus, Japan; Nikon Eclipse TE2000-E, Nikon, Japan).

Tissue sections from the ipsilateral lumbar spinal cord segments were randomly selected from each animal and were analyzed using a computer-assisted image analysis system (Metamorph version 7.7.2.0; PA, USA). Spinal cord dorsal horn was divided into three regions; superficial dorsal horn (SDH, laminae I and II), nucleus proprius (NP, laminae III and IV), and neck region (NECK, laminae V and VI). The positive pixel area of D-serine immunoreactivity was counted on % threshold area [(positive pixel area/pixel area in each region) × 100] in each region per section from each animal. To analyze the extent of colocalization of P450scc with GFAP (a marker of astrocytes), NeuN (a marker of neurons), or Iba-1 (a marker of microglial cells), we counted the number of cells visualized as yellow pixels in merged images. Colocalization was quantitated in the three dorsal horn regions as described above.

### Data Presentation and Statistical Analysis

The statistical significances of differences were assessed using Prism 5.0 (Graph Pad Software, San Diego, USA). Data obtained from the behavioral tests were analyzed by repeated measures two-way analysis of variance, and data obtained from the Western blot assay and immunohistochemistry were analyzed by one-way analysis of variance. The Bonferroni’s multiple comparison test was used for *post-hoc* analysis. All data are expressed as the mean ± SEM, and *P*values less than 0.05 were considered statistically significant.

## Results

### Intrathecal Administration of AMG Suppresses the Development of Neuropathic Pain, but Not Developed Pain in CCI Mice

**Figure 1:**
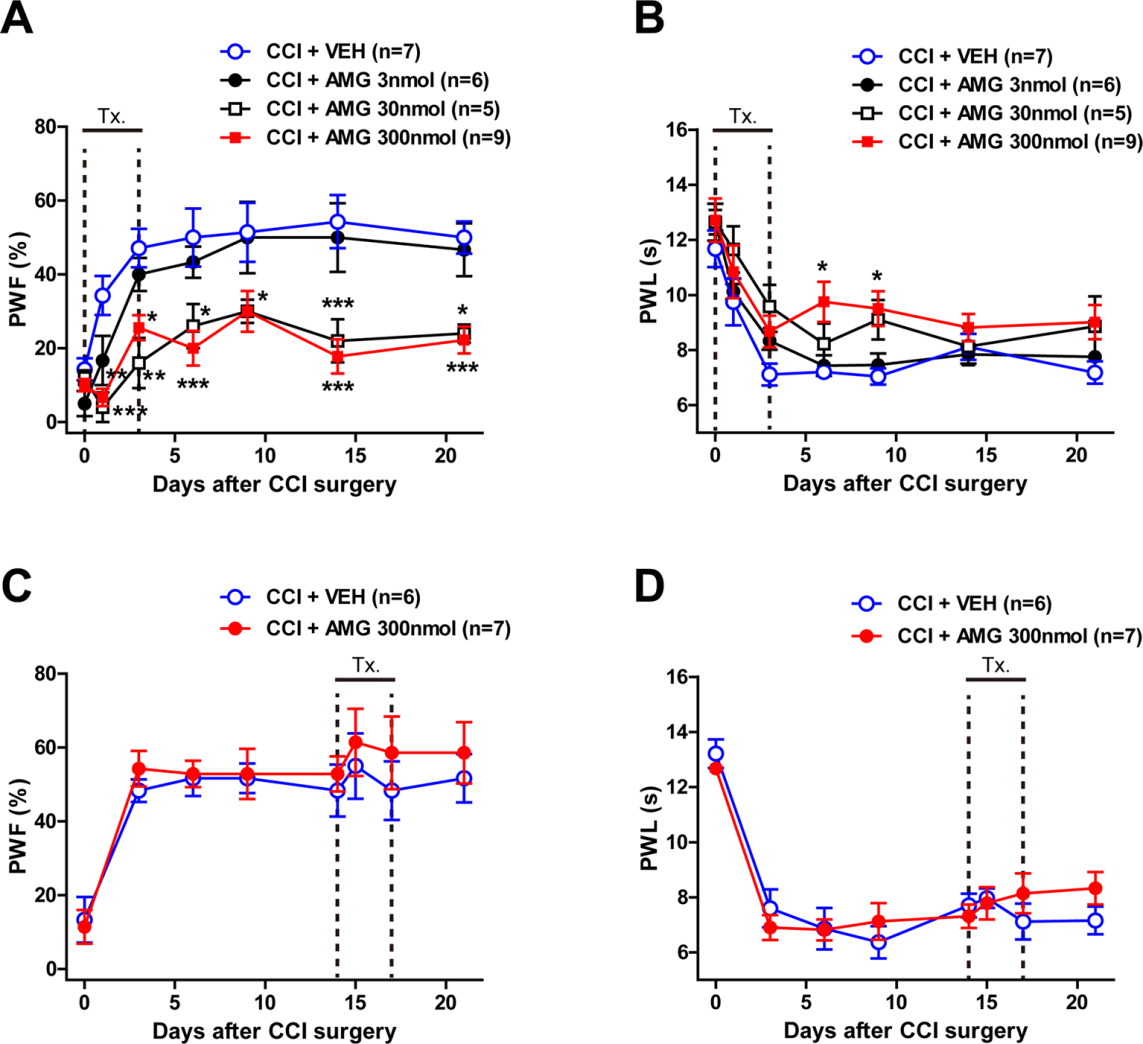
Graphs illustrating the effect of intrathecal (i.t.) administration of the P450scc inhibitor, aminoglutethimide (AMG), on neuropathic pain in chronic constriction injury (CCI) mice. **(A**–**D)** Paw withdrawal frequency (PWF, %) was measured in the hind paw using a von Frey filament (0.16 g), and paw withdrawal latency (PWL, s) was measured in the hind paw using a plantar analgesia meter. Administration of AMG (3, 30, 300 nmol) during the induction phase of neuropathic pain (from days 0 to 3 post-surgery) dose-dependently attenuated the CCI-induced development of mechanical allodynia **(A)** and thermal hyperalgesia **(B)**. On the other hand, administration of AMG (300 nmol) during the maintenance phase of neuropathic pain (from days 14 to 17 post-surgery) had no effect on the developed mechanical allodynia **(C)** and thermal hyperalgesia **(D)**. n = 5–9 mice/group. *P < 0.05, **P < 0.01, ***P < 0.001 vs. VEH-treated group.

To verify whether P450scc activation in the spinal cord is involved in the CCI-induced neuropathic pain, we intrathecally injected the P450scc inhibitor, AMG, during the induction phase and maintenance phase of neuropathic pain. CCI increased the paw withdrawal frequency (PWF, %) to innocuous mechanical stimuli and decreased the paw withdrawal response latency (PWL, s) to noxious heat stimulation (**Figure 1**). Intrathecal administration of AMG (3, 30, and 300 nmol) during the induction phase of neuropathic pain (days 0 to 3 post-surgery) dose-dependently attenuated both the CCI-induced development of mechanical allodynia (**Figure 1A**; group: *F*_3,161_ = 36.77, *P* < 0.0001; time: *F*_6,161_ = 17.60, *P* < 0.0001; interaction: *F*_18,161_ = 1.551, *P* = 0.0792) and thermal hyperalgesia (**Figure 1B**; group: *F*_3,161_ = 10.69, *P* < 0.0001; time: *F*_6,161_ = 26.23, *P* < 0.0001; interaction: *F*_18,161_ = 0.6662, *P* = 0.8404) as compared with vehicle (VEH)-treated CCI mice (**Figures 1A, B**; *P < 0.05, **P < 0.01, ***P < 0.001 vs. VEH-treated group).By contrast, i.t. administration of AMG (300 nmol) during the maintenance phase of neuropathic pain (days 14 to 17 post-surgery) had no effect on the developed mechanical allodynia (**Figure 1C**; group: *F*_1,88_ = 1.673, *P* = 0.1993; time: *F*_7,88_ = 9.668, *P* < 0.0001; interaction: *F*_7,88_ = 0.1719, *P* = 0.9902) and thermal hyperalgesia (**Figure 1D**; group: *F*_1,88_ = 0.2465, *P* = 0.6208; time: *F*_7,88_ = 26.65, *P* < 0.0001; interaction: *F*_7,88_ = 0.8918, *P* = 0.5166).

### Colocalization of P450scc With GFAP in the Spinal Cord Dorsal Horn Is Increased Following CCI

**Figure 2:**
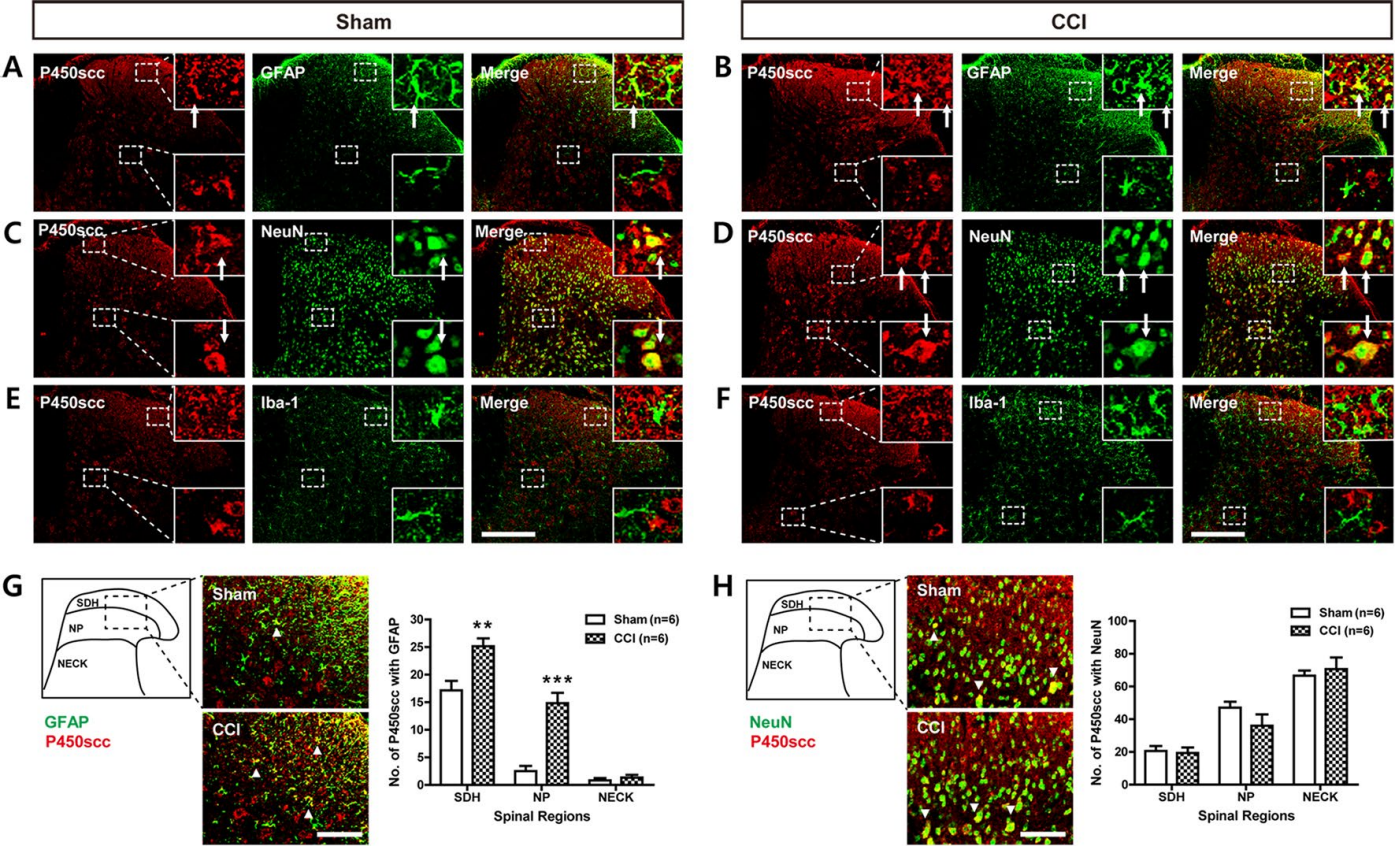
Images showing the cellular localization of P450scc in the lumbar spinal cord dorsal horn of sham- and CCI-operated mice and a graph showing the changes in the cytochrome P450 side-chain cleavage enzyme (P450scc) immunoreactivity within glial fibrillary acidic protein (GFAP)– or neuronal nuclei (NeuN)–immunoreactive cells following CCI. **(A**–**F)** Using a double-labeling immunohistochemical approach, these representative images depict colocalization (yellow) of P450scc with GFAP (**A** and **B**; green, a marker of astrocytes) or NeuN (**C** and **D**; green, a marker of neurons), but not with ionized calcium-binding adaptor molecule 1 (Iba-1) (**E** and **F**; green, a marker of microglial cells) at day 3 post-surgery in the lumbar spinal cord dorsal horn of sham- and CCI-operated mice. The arrows of higher magnification images depict examples of colocalization of P450scc with GFAP **(A** and **B)** and NeuN **(C** and **D)**. Scale bar = 200 μm. **(G** and **H)** Colocalization was quantitated in the superficial dorsal horn (SDH, laminae I–II), nucleus proprius (NP, laminae III–IV), and neck region (NECK, laminae V–VI). The number of P450scc-immunostained GFAP-positive cells **(G)** was increased in the SDH and NP regions following CCI of the sciatic nerve, while the number of P450scc-immunostained NeuN-positive cells **(H)** did not change following CCI. The arrowheads of images depict examples of colocalization of P450scc with GFAP **(G)** and NeuN **(H)**. Scale bar = 50 μm. n = 6 mice/group. **P < 0.01, ***P < 0.001 vs. sham.

To determine the cellular localization of P450scc in the spinal cord dorsal horn of sham- and CCI-operated mice, double immunohistochemistry was performed at day 3 post-surgery. P450scc immunoreactivity was colocalized with GFAP immunoreactivity in the SDHs of sham- and CCI-operated mice (**Figures 2A, B**) and with NeuN immunoreactivity in the superficial and deep dorsal horns of sham- and CCI-operated mice (**Figures 2C, D**), whereas there was no colocalization of P450scc with Iba-1 immunoreactivity (**Figures 2E, F**). In addition, the number of P450scc-immunostained astrocytes was significantly increased in the SDH (laminae I–II) and NP (laminae III–IV) regions following CCI [**Figure 2G**; **P < 0.01, ***P < 0.001 vs. sham; SDH: t(10) = 3.606, *P* = 0.0048; NP: t(10) = 5.828, *P* = 0.0002; NECK: t(10) = 0.7851, *P* = 0.4506]. By contrast, the number of P450scc-immunostained neurons did not change by CCI [**Figure 2H**; SDH: t(10) = 0.2552, *P* = 0.8037; NP: t(10) = 1.393, *P* = 0.1940; NECK: t(10) = 0.5195, *P* = 0.6147].

### Intrathecal Administration of AMG Given During the Induction Phase Inhibits Spinal Srr Expression in CCI Mice

**Figure 3:**
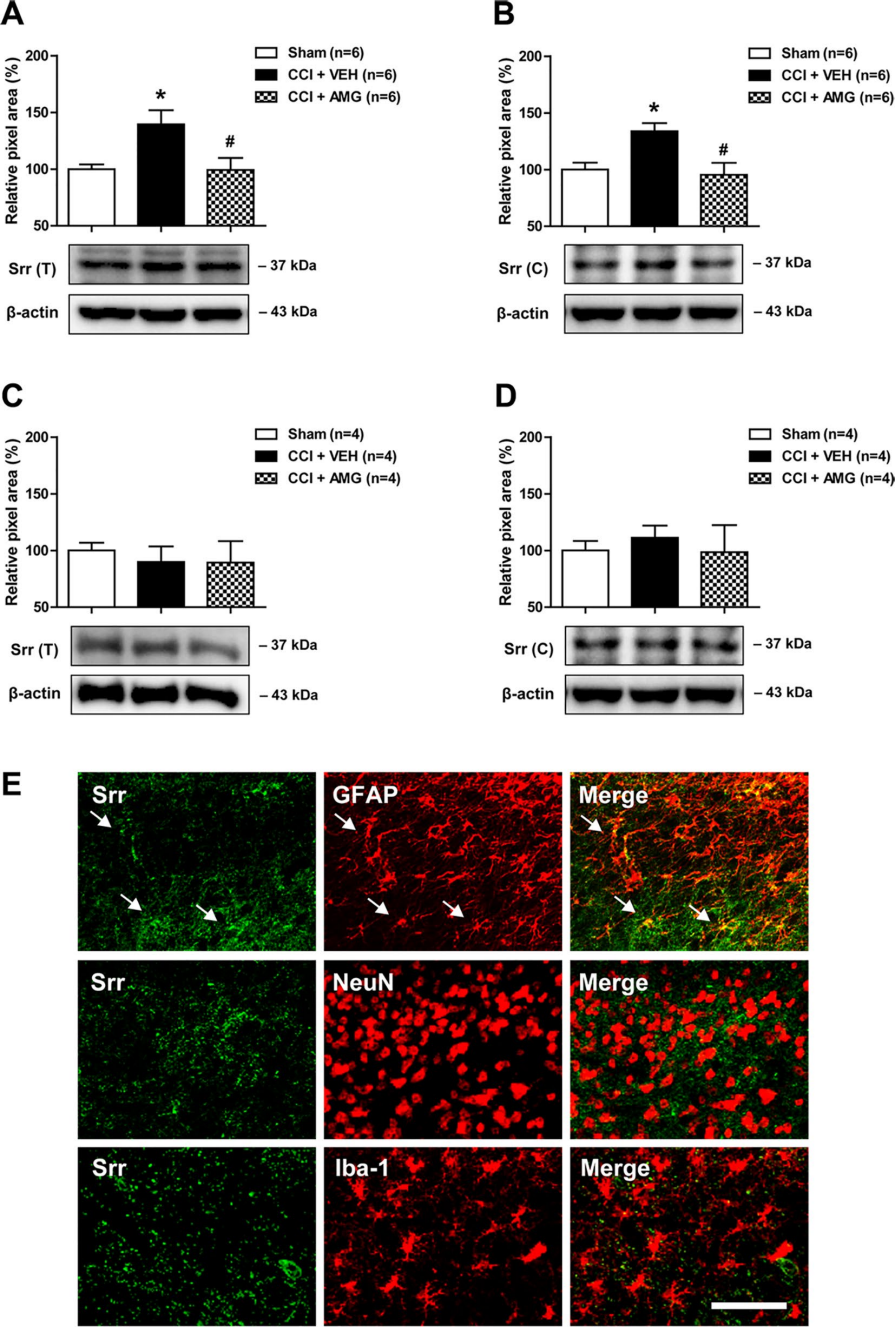
Western blots and graphs showing the effect of i.t. administration of the P450scc inhibitor, AMG, on serine racemase (Srr) expression in both the total (T) and cytosol (C) fractions of the lumbar spinal cord dorsal horn in CCI mice. **(A** and **B)** The graphs depicting the changes in the protein expression of Srr in the lumbar spinal cord dorsal horn are shown in the upper portion, while representative immunoblots are presented in the lower portion. Administration of AMG (300 nmol) given during the induction phase (from days 0 to 3 post-surgery) suppressed the CCI-induced increase in Srr expression in both the total **(A)** and cytosol **(B)** fractions. The spinal cord dorsal horn was sampled at 3 days after surgery. n = 6 mice/group. **(C** and **D)** By contrast, the total **(C)** and the cytosol **(D)** protein expression of Srr at day 17 post-surgery did not show any change after CCI. Administration of AMG (300 nmol) given during the maintenance phase (from days 14 to 17 post-surgery) had no effect on the Srr expression. The spinal cord dorsal horn was sampled at 17 days after surgery. n = 4 mice/group. *P < 0.05 vs. sham; ^#^ *P* < 0.05 vs. VEH-treated group. **(E)** Using a double-labeling immunohistochemical approach, these representative images depict colocalization (yellow) of Srr (green) with GFAP (red, a marker of astrocytes), but not with NeuN (red, a marker of neurons) or Iba-1 (red, a marker of microglial cells) at day 3 post-surgery in the lumbar spinal cord dorsal horn of CCI mice. The arrows in the first row of images depict examples of colocalization of Srr with GFAP in astrocytes. Scale bar = 50 μm.

In order to investigate the potential role of spinal P450scc on the expression and/or activation of Srr, a D-serine synthesizing enzyme, we examined the total (T) expression of spinal Srr, either the active form of Srr in their functional state at the cytosol (C) using a Western blot assay. CCI increased Srr expression in both the total (**Figure 3A**) and cytosol (**Figure 3B**) fractions of the lumbar spinal cord dorsal horn at day 3 after surgery (*P < 0.05 vs. sham). Intrathecal administration of AMG (300 nmol) during the induction phase of neuropathic pain (days 0 to 3 post-surgery) significantly suppressed this increase in both the total [**Figure 3A**; *F* (2,15) = 5.607, *P* = 0.0152] and cytosol [**Figure 3B**; *F* (2,15) = 6.605, *P* = 0.0088] fractions (#*P* < 0.05 vs. VEH-treated group). By contrast, there was no change in the Srr expression in both the total (**Figure 3C**) and cytosol (**Figure 3D**) fractions of the lumbar spinal cord dorsal horn at day 17 post-surgery when compared to the sham group. Administration of AMG (300 nmol) during the maintenance phase of neuropathic pain (days 14 to 17 post-surgery) had no effect on the Srr expression in the total [**Figure 3C**; *F* (2,9) = 0.1825, *P* = 0.8362] and cytosol [**Figure 3D**; *F* (2,9) = 0.1911, *P* = 0.8293] fractions as compared with the vehicle-treated group. Srr immunoreactivity was colocalized with GFAP immunoreactivity in the spinal cord dorsal horn of CCI mice, whereas there was no colocalization of Srr with NeuN or Iba-1 immunoreactivity (**Figure 3E**).

### Intrathecal Administration of AMG Given During the Induction Phase Inhibits Spinal D-Serine Immunoreactivity in CCI Mice

**Figure 4:**
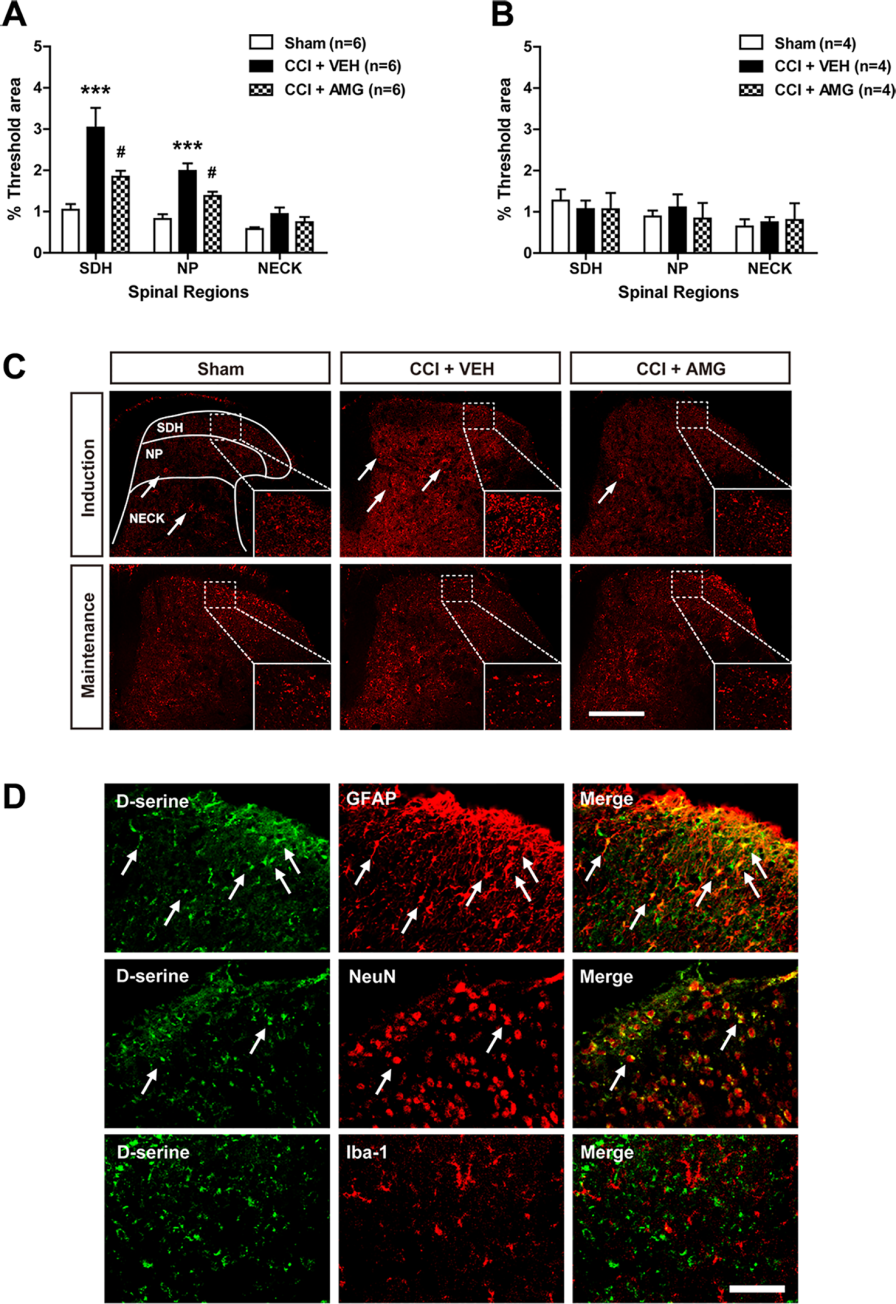
Graphs and photomicrographs illustrating the effect of i.t. administration of the P450scc inhibitor, AMG, on D-serine immunostaining in the lumbar spinal cord dorsal horn of CCI mice. **(A)** The fluorescence of D-serine immunoreactivity was quantitated in the SDH (laminae I–II), NP (laminae III–IV), and NECK (laminae V–VI) of mice. CCI increased D-serine immunoreactivity in the SDH and NP regions of the lumbar spinal cord dorsal horns at day 3 post-surgery. Administration of AMG (300 nmol) given during the induction phase (from days 0 to 3 post-surgery) suppressed the CCI-induced increase in D-serine expression. The spinal cord dorsal horn was sampled at 3 days after surgery. n = 6 mice/group. ***P < 0.001 vs. sham; ^#^ *P* < 0.05 vs. VEH-treated group. **(B)** By contrast, D-serine immunoreactivity at day 17 post-surgery did not show any change after CCI and administration of AMG (300 nmol) given during the maintenance phase (from days 14 to 17 post-surgery) had no effect on D-serine expression. The spinal cord dorsal horn was sampled at 17 days after surgery. n = 4 mice/group. **(C)** Representative images showing the changes in D-serine immunoreactivity at post-operative day 3 and 17 in the lumbar spinal cord dorsal horns of CCI mice using immunohistochemistry. Arrows indicate D-serine–immunoreactive cells. Scale bar = 200 μm. **(D)** Using a double-labeling immunohistochemical approach these representative images depict colocalization (yellow) of D-serine (green) with GFAP (red, a marker of astrocytes) or NeuN (red, a marker of neurons), but not with Iba-1 (red, a marker of microglial cells) at day 3 post-surgery in the lumbar spinal cord dorsal horn of CCI mice. The arrows in the first and second rows of images depict examples of colocalization of D-serine with GFAP and NeuN, respectively. Scale bar = 50 μm.

We next examined changes in D-serine immunoreactivity in the lumbar spinal cord dorsal horn using immunohistochemical analysis. CCI significantly increased D-serine immunoreactivity in the SDH (laminae I–II) and NP (laminae III–IV) regions of the spinal cords at day 3 post-surgery (**Figure 4A**; ***P < 0.001 vs. sham), and this increase was suppressed by i.t. administration of AMG (300 nmol) during the induction phase of neuropathic pain (days 0 to 3 post-surgery) [**Figure 4A**; #*P* < 0.05 vs. VEH-treated group; SDH: *F* (2,15) = 11.17, *P* = 0.0011; NP: *F* (2,15) = 18.01, *P* = 0.0001; NECK: *F* (2,15) = 0.0408, *P* = 0.1276]. By contrast, there was no change in D-serine immunoreactivity at day 17 post-surgery when compared to the sham group (**Figure 4B**). Administration of AMG (300 nmol) during the maintenance phase of neuropathic pain (days 14 to 17 post-surgery) had no effect on D-serine immunoreactivity at day 17 post-surgery as compared with vehicle-treated group [**Figure 4B**; SDH: *F* (2,9) = 0.1617, *P* = 0.8531; NP: *F* (2,9) = 0.09139, *P* = 0.9135; NECK: *F* (2,9) = 0.2312, *P* = 0.7982]. Representative photomicrographs of L_4–5_ spinal cord sections illustrating D-serine immunoreactivity in the sham group, vehicle-treated CCI group, and AMG-treated group are shown in **Figure 4C**. D-serine immunoreactivity was colocalized with GFAP and NeuN immunoreactivity in the spinal cord dorsal horn of CCI mice, whereas there was no colocalization of D-serine with Iba-1 immunoreactivity (**Figure 4D**).

### Co-Administration of D-Serine With AMG Given During the Induction Phase Restores the Development of Mechanical Allodynia in CCI Mice

**Figure 5:**
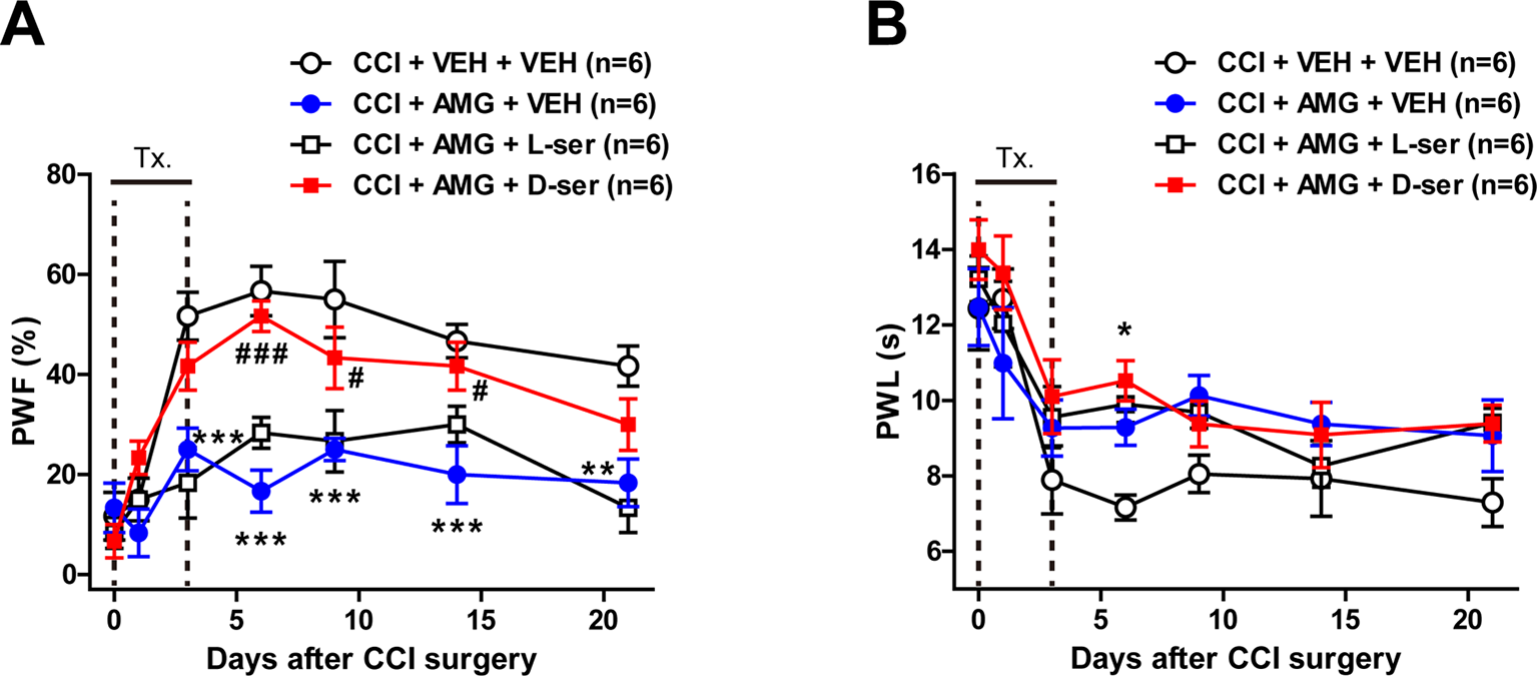
Graphs illustrating the effects of concomitant D-serine and AMG treatment on the development of neuropathic pain in CCI mice. **(A** and **B)** PWF (%) was measured in the hind paw using a von Frey filament (0.16 g), and PWL (s) was measured in the hind paw using a plantar analgesia meter. Intrathecal administration of AMG (300 nmol) given during the induction phase (from days 0 to 3 post-surgery) attenuated the CCI-induced development of mechanical allodynia **(A)** and thermal hyperalgesia **(B)**. Co-administration of exogenous D-serine (500 nmol) with AMG restored the CCI-induced mechanical allodynia that was initially blocked by AMG administration **(A)**. On the other hand, co-administration of D-serine (500 nmol) with AMG had no effect on the CCI-induced development of thermal hyperalgesia **(B)**. Similarly, co-administration of D-serine’s enantiomer, L-serine (500 nmol), with AMG had no effect on the CCI-induced development of mechanical allodynia **(A)** and thermal hyperalgesia **(B)** that were initially blocked by AMG administration alone. n = 6 mice/group. *P < 0.05, **P < 0.01, ***P < 0.001 vs. VEH+VEH-treated group; ^#^ *P* < 0.05, ^###^ *P* < 0.001 vs. AMG+VEH-treated group.

To confirm the potential role of D-serine in the development of neuropathic pain induced by activation of P450scc in CCI mice, D-serine was co-administrated with AMG during the induction phase of neuropathic pain (days 0 to 3 post-surgery). Intrathecal administration of AMG (300 nmol) inhibited the CCI-induced increase in the PWF (%) to innocuous mechanical stimuli and the CCI-induced decrease in the PWL (s) to noxious heat stimulation (**Figure 5**; *P < 0.05, **P < 0.01, ***P < 0.001 vs. VEH-treated group). Co-administration of D-serine (500 nmol) with AMG restored the CCI-induced development of mechanical allodynia that was inhibited by AMG administration alone (**Figure 5A**; #*P* < 0.05, ###*P* < 0.001 vs. AMG+VEH-treated group; group: *F*_3,140_ = 35.39, *P* < 0.0001; time: *F*_6,140_ = 22.96, *P* < 0.0001; interaction: *F*_18,140_ = 2.717, *P* = 0.0005). By contrast, co-administration of D-serine (500 nmol) with AMG had no effect on the CCI-induced development of thermal hyperalgesia that was inhibited by AMG administration alone (**Figure 5B**; group: *F*_3,140_ = 6.236, *P* = 0.0005; time: *F*_6,140_ = 20.88, *P* < 0.0001; interaction: *F*_18,140_ = 0.8239, *P* = 0.6698). Co-administration of D-serine’s enantiomer, L-serine (500 nmol), with AMG had no effect on the development of mechanical allodynia (**Figure 5A**) or thermal hyperalgesia (**Figure 5B**) that was inhibited by AMG administration alone in CCI mice.

## Discussion

Neurosteroids are locally synthesized in the central nervous system and are involved in modulating a number of neural functions including nociception (Yoon et al., 2010; Joksimovic et al., 2018). It has been suggested that direct administration of neurosteroids regulates many pathophysiological responses, but use of neurosteroids to treat epilepsy, neuropsychiatric disorders, pain, and other neurologic issues has been met with several challenges, including pharmacokinetics, low bioavailability, safety, and tolerability, which limit its therapeutic use. Therefore, modulation of neurosteroidogenesis to restore the altered endogenous neurosteroid tone may represent a better therapeutic approach than direct injection of neurosteroids (Porcu et al., 2016). In previous studies from our laboratories, we have shown that activation of P450c17 plays an important role in the spinal nociceptive signal transmission following peripheral neuropathy, and that early increased pro-inflammatory cytokine interleukin-1β controls the expression of P450c17 in the spinal cord astrocytes of CCI mice (Choi et al., 2019b; Choi et al., 2019c). However, there is limited understanding of the potential role of P450scc, which catalyzes the first rate-limiting step in neurosteroid biosynthesis, in neuropathic pain. In the present study, our findings demonstrate for the first time that spinal P450scc activation plays an important role in the increases in astrocyte Srr expression and accompanying D-serine production in a murine model of neuropathic pain, ultimately contributing to the development of mechanical allodynia in CCI mice.

It has been suggested that glial cells play a prominent role in the local production of neurosteroids and in the mediation of neurosteroid effects on neurons and glial cells (García-Estrada et al., 1999; Garcia-Segura and Melcangi, 2006). In the present study, P450scc immunoreactivity in the spinal cord of CCI-operated mice was increased in GFAP-positive astrocytes, while there was no change in P450scc expression in NeuN-positive neurons, suggesting the possibility that the conversion of cholesterol to PREG catalyzed by P450scc is increased in GFAP-positive astrocytes following peripheral neuropathy. In addition, i.t. administration of the P450scc inhibitor, AMG, during the early phase of neuropathic pain significantly reduced the CCI-induced increase in Srr expression in both the total and cytosol fractions of the spinal cord. Since the cytosolic Srr is largely active, exhibiting about 10 times of the specific activity observed with the membrane-bound enzyme (Balan et al., 2009), a decrease in the cytosol fraction of Srr has been considered to represent a decrease in the active form of the Srr. These results suggest the possibility that increased neurosteroidogenesis initiated by astrocyte P450scc during the induction phase of neuropathic pain plays an important role in the modulation of Srr activation and expression in astrocytes following peripheral nerve injury.

There are two potential mechanisms by which Srr expression may be modulated by spinal P450scc activation. It is generally accepted that neurosteroids can mediate their actions, not only through classic steroid hormone nuclear receptors leading to regulation of gene expression, but also through other mechanisms such as the direct or indirect modulation of membrane-bound receptors including NMDA (Baulieu, 1997; Compagnone and Mellon, 2000), γ-aminobutyric acid type A (GABA_A_) (Haage and Johansson, 1999), and sigma-1 receptors (Ueda et al., 2001; Maurice, 2004). These receptors play an important role in neuronal excitability and nociceptive signal transmission in the spinal cord dorsal horn (Qu et al., 2009; Gwak and Hulsebosch, 2011; Castany et al., 2019). In a previous study from our laboratories, we showed that sigma-1 receptors are exclusively expressed in astrocytes and co-localized with Srr immunoreactivity in the spinal cord dorsal horn (Moon et al., 2014; Moon et al., 2015). The activation of this receptor increases Srr expression and concomitant D-serine production following peripheral nerve injury (Moon et al., 2015). Since neurosteroids such as PREG-sulfate (PREG-S) and DHEA-sulfate (DHEA-S) activate sigma-1 receptors *via* agonistic stimulation (Hayashi and Su, 2007), P450scc-induced neurosteroidogenesis may modulate the expression of Srr through a sigma-1 receptor–dependent pathway. In addition, it has been suggested that PREG-S and DHEA-S are allosteric inhibitors of the GABA_A_ receptor (Sachidanandan and Bera, 2015); thus, neurosteroids synthesized by activation of P450scc may also decrease GABAergic inhibitory signaling in astrocytes. Although these results suggest the possibility that neurosteroidogenesis induced by sciatic nerve injury may increase astrocyte Srr expression *via* regulation of steroid hormone nuclear receptors and/or membrane-bound receptors, the specific details of the underlying mechanisms remain to be elucidated.

D-serine is an endogenous co-agonist for the glycine site on the NMDA receptors and contributes to NMDA receptor–mediated neurotransmission (Mothet et al., 2000). In the present study, early inhibition of P450scc significantly inhibited the CCI-induced increase in D-serine production, which was primarily increased in the SDH (laminae I–II) and NP (laminae III–IV) regions in the spinal cord following CCI. These results are supported by a study demonstrating that the expression of the cytochrome P450scc increases in the SDH region and this increase spreads into the NP region of the cord following sciatic nerve ligation (Patte-Mensah et al., 2006). Since primary afferent C-fibers and A-fibers terminate and synapse with second-order neurons in the SDH and NP regions (Todd, 2010), the current results suggest the possibility that CCI-induced early activation of P450scc may play a critical role in the dorsal horn transmission of nociceptive signaling at least partially through modulation of D-serine production. In addition, we previously demonstrated that D-serine plays an important role in the functional potentiation of NMDA receptors *via* increases in phosphorylation of the GluN1 subunit (Choi et al., 2017), which is known to be an essential contributor to the process of central sensitization (Kim et al., 2006; Roh et al., 2008). While D-serine’s modulation of nociceptive transmission may contribute to the induction of central sensitization and the development of mechanical allodynia following peripheral nerve injury, the CCI-induced development of thermal hyperalgesia appears to be associated with different mechanisms, which are P450scc-mediated but not D-serine–dependent.

NMDA receptors have three major subunits: GluN1, GluN2 (A–D), and GluN3 (A or B) (Traynelis et al., 2010). While GluN1 and GluN3 bind D-serine or glycine, GluN2 binds glutamate. Activation of GluN2-containing receptors requires both D-serine or glycine and glutamate, whereas receptors composed of GluN1 and GluN3 subunits can be activated by D-serine or glycine in the absence of glutamate (Chatterton et al., 2002; Awobuluyi et al., 2007; Balasuriya et al., 2014). In the spinal cord, GluN1 is expressed in all laminae of the spinal cord, and GluN2B is mostly distributed in the SDH (Qu et al., 2009). In a previous study from our laboratories, we showed that sciatic nerve injury increases the expression of GluN1 subunit in the spinal cord dorsal horn during the induction phase of neuropathic pain (Roh et al., 2008). Furthermore, it has been suggested that i.t. administration of GluN2B antagonists decreases not only the C-fiber responses of dorsal horn wide dynamic range neurons, but also nerve injury–induced mechanical allodynia (Qu et al., 2009). While RNA sequencing data revealed a high level of GluN3 messenger RNAs (mRNAs) in the dorsal root ganglia, the physiological and pathological roles of GluN3 subunits in regulating nociception remains unclear. In the present study, co-administration of exogenous D-serine with AMG during the induction phase of neuropathic pain restored the development of mechanical allodynia that was originally suppressed by inhibition of P450scc. Exogenous D-serine may activate NMDA receptors *via* binding to GluN1 and/or GluN3 subunits, but this should be further investigated. On the other hand, i.t. administration of L-serine, the enantiomer of D-serine, had no effect on the CCI-induced neuropathic pain. Since L-serine is inactive and should be metabolized to D-serine by Srr, AMG-induced inhibition of Srr expression and activation would suppress the metabolism of L-serine to D-serine, thus reducing this conversion and the potential effects of D-serine from this metabolic pathway.

In the present study, inhibition of P450scc during the induction phase (days 0 to 3 post-surgery) significantly reduces the development of neuropathic pain, while inhibition of P450scc during the maintenance phase (days 14 to 17 post-surgery) had no effect on the later stages of neuropathic pain. These results raise the possibility that the net effect of neurosteroidogenesis is pro-nociceptive during the early phase, but without effect during the late phase of neuropathic pain. In this regard, it has been shown that PREG, which is synthesized by P450scc, can be catalyzed by cytochrome P450c17 and converted to DHEA. DHEA is then capable of producing rapid pronociceptive effects (Kibaly et al., 2008). Furthermore, PREG can be converted to progesterone and its reduced metabolite allopregnanolone, which stimulate GABA_A_ receptors and induce analgesia (Meyer et al., 2008). Thus, the net effect of neurosteroidogenesis appears to be dependent on the changes in downstream enzyme activity. This is supported by a previous study showing that the level of cytochrome P450c17 mRNA in the rat spinal cord is significantly decreased at 10 days post–CCI surgery (Kibaly et al., 2008), while the enzymatic activity of 3α-hydroxysteroid oxidoreductase, an allopregnanolone synthesizing enzyme, is increased in the spinal cord at 10 days post–CCI surgery in rats (Meyer et al., 2008). Meyer and colleagues also demonstrated that inhibition of 3α-hydroxysteroid oxidoreductase during this maintenance phase potentiated the neuropathic pain following CCI and suggested that a large portion of neurosteroidogenesis that occurs during the maintenance phase may constitute an endogenous control mechanism to overcome the chronic pain state induced by peripheral nerve injury (Meyer et al., 2008). Furthermore, recent findings suggest that descending pain facilitatory and inhibitory circuits are likely to be an important element in determining whether pain may become chronic (Ossipov et al., 2014). Abnormal activation of glial cells in the spinal cord also plays important role in the maintenance of chronic pain (Gwak and Hulsebosch, 2011). In this regard, we plan to investigate the detailed mechanisms underlying the maintenance of chronic neuropathic pain in future studies.

In conclusion, the present study shows that sciatic nerve injury significantly increases the expression of P450scc in spinal astrocytes, but not neurons, during the induction phase of neuropathic pain, and that early inhibition of P450scc with AMG significantly suppresses the development of neuropathic pain following peripheral nerve injury. Moreover P450scc modulates the expression and activation of astrocyte Srr and the concomitant production of D-serine that potentiates NMDA receptor–mediated signaling, and ultimately contributes to the development of mechanical allodynia following peripheral neuropathy. This study suggests the potential therapeutic use of P450scc inhibitors to prevent or modulate the development of peripheral neuropathic pain.

## Data Availability Statement

The data sets for this manuscript are not publicly available because of security issues. Requests to access the data sets should be directed to J-HL, jhl1101@snu.ac.kr.

## Ethics Statement

The experimental protocols for animal usage were reviewed and approved by the SNU Institutional Animal Care and Use Committee following the National Institutes of Health guide for the care and use of laboratory animals (NIH Publications No. 96-01) revised in 1996.

## Author Contributions

Study concept and design: S-RC, J-HL. Acquisition, analysis, and interpretation of data: S-RC, AB, J-HL. Drafting of the article: S-RC, AB, J-HL. Final approval of the version to be submitted: J-HL.

## Funding

This work was supported by the National Research Foundation of Korea (NRF) grant funded by the Korean Government (grant number 2017R1A2A2A05001402).

## Conflict of Interest

The authors declare that the research was conducted in the absence of any commercial or financial relationships that could be construed as a potential conflict of interest.
